# Antioxidants as Adjuvants in Periodontitis Treatment: A Systematic Review and Meta-Analysis

**DOI:** 10.1155/2019/9187978

**Published:** 2019-07-22

**Authors:** Micaele Maria Lopes Castro, Nathallia Neves Duarte, Priscila Cunha Nascimento, Marcela Barauna Magno, Nathalia Carolina Fernandes Fagundes, Carlos Flores-Mir, Marta Chagas Monteiro, Cassiano Kuchenbecker Rösing, Lucianne Cople Maia, Rafael Rodrigues Lima

**Affiliations:** ^1^Laboratory of Functional and Structural Biology, Institute of Biological Sciences, Federal University of Pará, Belém, Brazil; ^2^Department of Pediatric Dentistry and Orthodontics, School of Dentistry, Federal University of Rio de Janeiro, Rio de Janeiro, Brazil; ^3^School of Dentistry, Faculty of Medicine and Dentistry, University of Alberta, Edmonton, Canada; ^4^School of Pharmacy, Federal University of Pará, Belém, Pará, Brazil; ^5^Department of Periodontology, Faculty of Dentistry, Federal University of Rio Grande do Sul, Porto Alegre, Brazil

## Abstract

This systematic review with meta-analysis aimed to evaluate the effect of antioxidants as an adjuvant in periodontitis treatment. The following databases were consulted: PubMed, Scopus, Web of Science, Cochrane, Lilacs, OpenGrey, and Google Scholar. Based on the PICO strategy, the inclusion criteria comprised interventional studies including periodontitis patients (participants) treated with conventional therapy and antioxidants (intervention) compared to patients treated only with conventional therapy (control) where the periodontal response (outcome) was evaluated. The risk of bias was evaluated using the Cochrane RoB tool (for randomized studies) and ROBINS-I tool (for nonrandomized studies). Quantitative data were analyzed in five random effects meta-analyses considering the following periodontal parameters: clinical attachment loss (CAL), plaque index (PI), gingival index (GI), bleeding on probing (BOP), and probing depth (PD). After all, the level of certainty was measured with the Grading of Recommendation, Assessment, Development, and Evaluation (GRADE) tool. Among the 1884 studies identified, only 15 interventional studies were according to the eligibility criteria and they were included in our review. From them, 4 articles presented a high risk of bias. The meta-analysis showed a statistically significant difference for CAL (SMD 0.29 (0.04, 0.55), *p* = 0.03, *I*^2^ = 13%), PI (SMD 0.41 (0.18, 0.64), *p* = 0.0005, *I*^2^ = 47%), and BOP (SMD 0.55 (0.27, 0.83), *p* = 0.0001, *I*^2^ = 0%). The GRADE tool showed a moderate to high certainty in the quality of evidence depending on the clinical parameter and antioxidants used. These results suggest that the use of antioxidants is an adjunct approach to nonsurgical periodontal therapy which may be helpful in controlling the periodontal status.

## 1. Introduction

Periodontal disease is a chronic inflammatory manifestation in the tissues surrounding the teeth caused by an imbalance between oral biofilms and the host's response, in which there is a possibility of loss of tooth support tissues [1, 2]. The state of being restricted to protective periodontium is characterized as gingivitis, and that when affecting the periodontal supporting tissues is called periodontitis [3]. Periodontitis is one of the most prevalent diseases of the oral cavity, caused by bacterial plaque microorganisms and influenced by factors such as systemic condition, oral hygiene, age, sex, and smoking [4].

The excessive presence of free radicals caused by oxidative stress or antioxidant deficiency has been linked to periodontal disease [5]. Early in the progression of periodontal disease, especially in periodontitis, there is a marked oxidative process with increased levels of reactive oxygen and nitrogen species (ROS and RNS). This process can lead to an imbalance in the body response, with concomitant changes in biomolecules, especially lipids, proteins, and nucleic acids, resulting in periodontal tissue damage [6].

The antioxidant defense system can inhibit and/or reduce the damage caused by deleterious action of free radicals or nonradical reactive species [7]. Some antioxidant sources found in nature (foods, teas, vitamins, minerals, among others) are currently used in various auxiliary treatments for cardiovascular diseases, pulmonary diseases, ageing, and atherosclerosis [8–11]. These conditions have physiologic links with periodontal diseases which would lead to the assumptions that a potential benefit could also be observed under these therapies.

In the search for adjuvants to conventional periodontal treatments which could present worse than expected results, some literature suggests that supplementation with antioxidant components may help to reduce periodontal damage and its systemic effects when compared to treatment with antibiotics that can cause resistance or onset of secondary infections [12]. There is not necessarily a consensus in this regard, as the evidence regarding the supporting role of antioxidant agents as adjuvants to periodontal treatment is limited which makes clinical decision-making difficult. Therefore, this systematic review is aimed at assessing whether antioxidants have some beneficial effect on the treatment of periodontitis.

## 2. Material and Methods

### 2.1. Focus Question and Registration

In order to perform this systematic review, the following question was elaborated: “In patients with periodontitis, do antioxidants have an additional clinically meaningful effect when used as adjuvants to conventional therapy?”

This systematic review was registered under the number CRD42017079869 in the PROSPERO database, created by the University of York, responsible for the registration and dissemination of systematic reviews and carried out according to the PRISMA (Preferred Reporting Items for Systematic Reviews and Meta-Analyses) statement [13].

### 2.2. Search Strategy and Eligibility Criteria

The PICO strategy was applied in this systematic review. This acronym stands for an abbreviation of the following components: patient, intervention, comparison, and outcome, which are essential for designing all stages of an interventional systematic review. We included intervention studies in adult humans with periodontitis (P) that compared conventional periodontal treatment with the addition of antioxidants (I) compared to patients who have undergone only conventional periodontal treatment (C) in order to observe periodontal treatment effects (O). Additionally, case reports, descriptive studies, review articles, opinion articles, technical articles, guidelines, animal studies, and *in vitro* studies were not considered.

The electronic searches were carried out in the following databases: PubMed, Scopus, Web of Science, Lilacs, Cochrane, Google Scholar, and OpenGrey. There were no restrictions on the date of publication or in the language used in the primary studies. The terms MeSH, keywords, and search strategies were adapted according to each database (Table 1). The searches were carried out up to February 2019.

A search alert was created in each database to notify new studies according to the outlined search strategy. All relevant citations were imported into a bibliographic reference manager (EndNote®, version X7, Thomson Reuters, Philadelphia, USA).

Through the EndNote® manager, the removal of duplicate articles was performed using automatic exclusion added to the manual deletion. After the exclusions, the texts were analyzed for the titles and abstracts and, afterwards, from the reading of the full text when indicated, according to the established eligibility criteria.

### 2.3. Data Extraction and Quality Assessment

The eligible articles resulting from the previous selection were extracted and tabulated including information of the country, year, characteristics of the participants (sample size and age), periodontal parameters measured, the antioxidant used, conventional treatment performed, results, and statistical analysis.

The risk of bias assessment was based on two tools. “The Cochrane Collaboration's tool for assessing risk of bias” tool [14] through the Review Manager software (version 5.3, Review Manager (RevMan) (computer program) version 5.3. Copenhagen: The Nordic Cochrane Centre, The Cochrane Collaboration, 2014) was used for randomized intervention studies and the ROBINS-I (Risk of Bias in Non-randomized Studies of Interventions) tool [15] for nonrandomized studies.

“The Cochrane Collaboration's tool for assessing risk of bias” tool [14] consists of a checklist of seven key areas: sequence generation randomized, concealment selection, blinding of participants, evaluation of blinding result, incomplete result data, selective reporting bias, and other risks (Table 2). For each study, the risk of bias was judged for each domain and the overall assessment as low risk, high risk, or uncertain risk for all included studies.

The ROBINS-I (Risk of Bias in Non-randomized Studies of Interventions) tool [15] is composed of three main domains for bias evaluation: preintervention, during intervention, and postintervention (Table 3). The risk of bias was judged for each domain and classified in its general assessment as low, moderate, serious, critical, or no information for all included studies.

All evaluations, including searches, study selection, and data extraction, were performed independently by two reviewers (NND and MMLC) and checked by a third-party evaluator (RRL) in case of disagreement.

### 2.4. *Meta-Analysis*

Data from the included studies were analyzed using Comprehensive Meta-Analysis software (version 3.2; Biostat) to evaluate the efficacy of periodontal therapy (PT) associated with antioxidants in periodontal parameters. This treatment protocol (PT plus antioxidants) was compared to conventional PT only. The main periodontal parameters analyzed by the studies were evaluated in five different random effects meta-analyses: (1^st^) clinical attachment loss (CAL), (2^nd^) plaque index (PI), (3^rd^) gingival index (GI), (4^th^) bleeding on probing (BOP), and (5^th^) probing depth (PD). The average and standard deviation of each parameter and the total number of individuals of each group (PT only and PT plus antioxidants) were used. A subgroup analysis was conducted considering the follow-up periods evaluated in studies.

As the studies reported the outcome using similar methods for all parameters, the standard mean difference (SMD) was applied [16], with 95% confidence interval (CI). Only studies considered as having “low,” “unclear,” and “moderate” risks of bias were included in the meta-analysis. If some of the information needed for the meta-analysis was absent from any of the selected studies, the authors were contacted to provide the missing data. Studies considered as having a high risk of bias and/or remaining without sufficient data for the quantitative analysis, after contact with the authors, were excluded from the meta-analysis.

Heterogeneity was tested using the *I*^2^ index, and if possible, sensitivity analyses were conducted to estimate and verify the influence of studies, one by one, on the pooled results, when the heterogeneity was substantial or considerable (50 to 100%) [16]. Random effects models were employed taking into consideration that the studies were not functionally equivalent in which the objective was to generalize the results from the meta-analysis [17].

### 2.5. Level of Evidence

A summary of the overall strength of evidence was presented using the “Grading of Recommendation, Assessment, Development, and Evaluation” (GRADE) tool [18]. Evidence from randomized clinical trials is initially classified as high quality, but the assurance on this evidence may be reduced for a number of reasons including the following: the methodological design, study quality, consistency, and directness. Three subgroups were created dividing according to the antioxidants used: (1) several antioxidants; (2) lycopene; (3) green tea.

## 3. Results

### 3.1. Characteristics of the Included Studies

**Figure 1:**
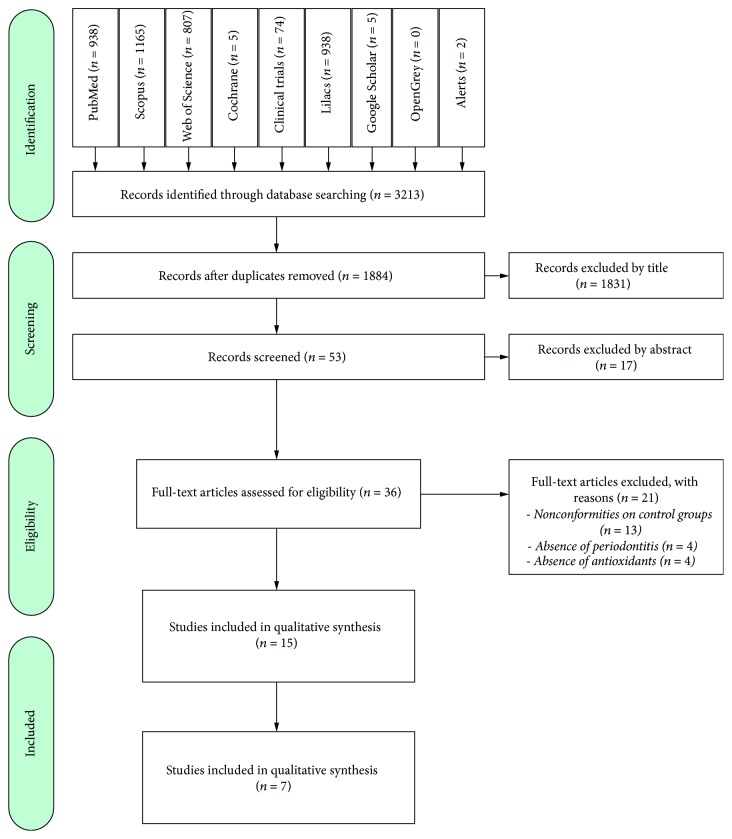
Flow diagram of literature search according to the PRISMA statement.

The searches in databases and gray literature articles amounted to 3213, resulting in 1884 after exclusion of duplicates. All 1884 remaining articles were analyzed by titles and abstracts, based on the eligibility criteria, and then 1831 were excluded. Thirty-six studies were analyzed in full text, and 21 of them were excluded, having 15 articles remaining that were included in this review to qualitative synthesis [19–33], and 7 of them were designated to quantitative synthesis [19, 22, 23, 25, 28, 30, 32] (Figure 1).

All studies evaluated periodontitis, and in those where there was a group for gingivitis and group for periodontitis presented in the same study [22, 29], only data referring to periodontitis were considered. Authors, study design, sample description, periodontitis diagnostic method, type of periodontal and antioxidant treatment, statistical analysis, and the outcome of each included article were described in Table 4.

The PD was the most used evaluation method, present in all studies, followed by gingival and plaque indexes (GI and PI, respectively), CAL, and BOP. One study reports the additional use of the community periodontal index (CPI) as an evaluation method [28]. Regarding periodontitis treatment, all studies carried out scaling and root planning; however, only one study added a surgical treatment, the modified Widman flap procedure, specifically at baseline visit.

Some studies have evaluated laboratory aspects besides clinical aspects such as markers of bone resorption (RANKL) [19], markers of inflammatory response (interleukins, tumor necrosis factor alpha, and Pentraxin-3) [20, 24, 26], nitrite/nitrate ratio, and antioxidant activity markers (total antioxidant capacity, glutathione-S-transferase, uric acid, and superoxide dismutase) [21, 23, 25, 29, 31, 32], present in saliva, crevicular fluid, or plasma. Only five studies did not perform laboratory tests [22, 27, 28, 30, 33].

### 3.2. Risk of Bias

**Figure 2:**
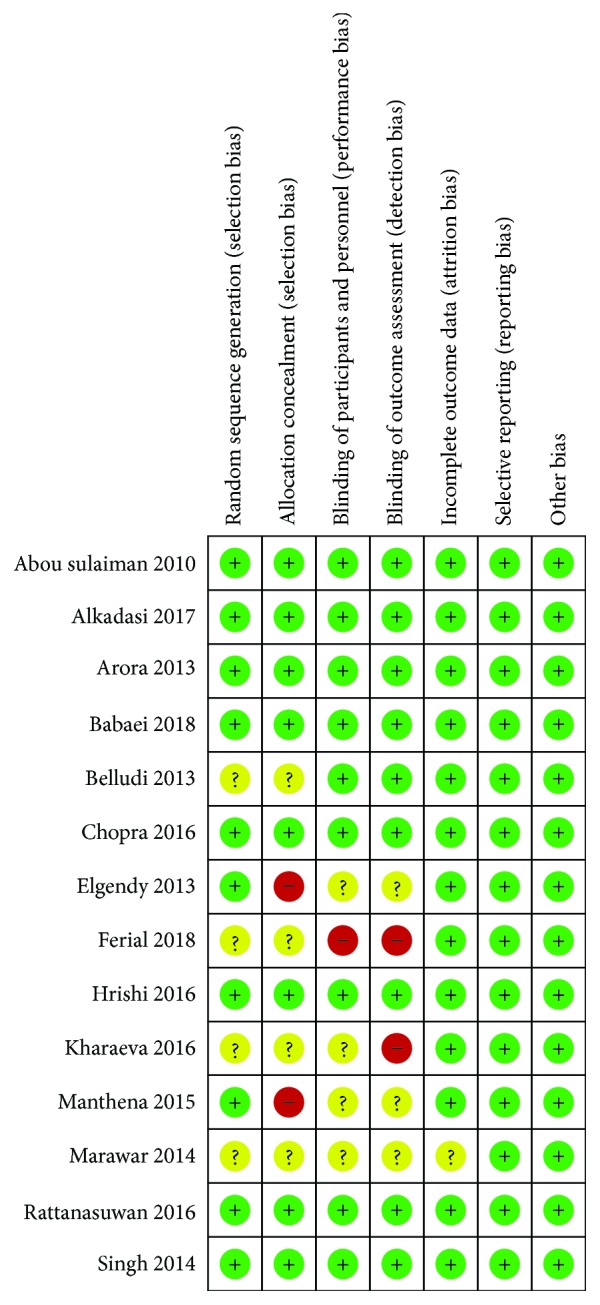
Risk of bias evaluation in randomized trials (Cochrane Collaboration's tool).

A total of 14 randomized clinical trials were assessed for bias risk using the Cochrane tool [14]. The key domains chosen for the high-risk bias trial were allocation concealment, blinding of participants and personnel, and blinding of outcome assessment. From them, 11 studies showed a low risk of bias while 4 studies demonstrated a high risk of bias from the evaluation proposed by the key domains chosen [24, 26, 27, 33] (Figure 2).

In the risk of bias analysis in the ROBINS-I model, the study by Mathur et al. [29] presented a low risk of bias in almost all domains, except in the bias of missing data, because it did not present enough data where some judgment could be attributed (Table 5).

### 3.3. *Meta-Analysis*

Four studies were excluded from the meta-analyses because they were classified as having “high” risk of bias [24, 26, 27, 33]. Data extracted by the Singh et al. study were imprecise, and for this reason, this study was excluded from the quantitative synthesis. The meta-analysis results were presented separately for each parameter.

#### 3.3.1. Meta-Analysis for Clinical Attachment Loss (CAL)

**Figure 3:**
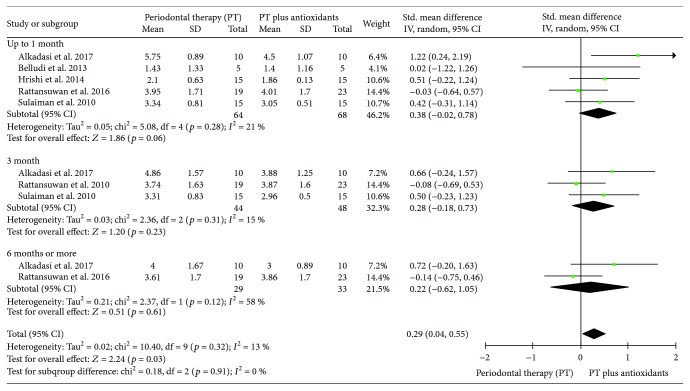
Forest plot of the first meta-analysis for clinical attachment loss (CAL).

Seven clinical trials evaluating the influence of PT additionally to antioxidant therapy in the mean of CAL were included in this analysis. Including all studies, the heterogeneity was *I*^2^ = 61%. During sensitivity analysis, the heterogeneity ranges from 20% to 66%, and in an attempt to reduce the overall and subgrouped heterogeneity, the study of Chopra et al. [23] was excluded from the final analysis. In subgroup analysis, independent of follow-up point evaluated, individuals treated with PT plus antioxidants presented a mean CAL, in millimeters, similar to individuals treated with PT only (up to one month (SMD 0.38 (-0.02, 0.78), *p* = 0.06, *I*^2^ = 21%, *I*^2^*p* = 0.28), three months (SMD 0.28 (-0.18, 0.73), *p* = 0.23, *I*^2^ = 15%, *I*^2^*p* = 0.31), or six months or more (SMD 0.22 (-0.62, 1.05), *p* = 0.61, *I*^2^ = 58%, *I*^2^*p* = 0.12)). However, in pooled results, individuals treated with PT plus antioxidants (*n* = 68) presented a mean CAL, in millimeters, lower than individuals treated with PT only (*n* = 64) (SMD 0.29 (0.04, 0.55), *p* = 0.03, *I*^2^ = 13%, *I*^2^*p* = 0.32; Figure 3).

#### 3.3.2. Meta-Analysis for the Plaque Index (PI)

**Figure 4:**
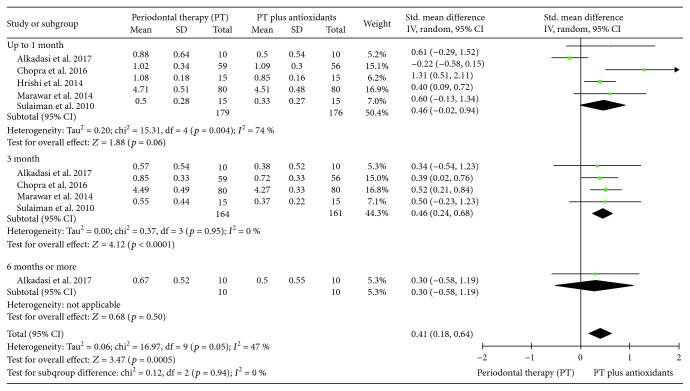
Forest plot of the second meta-analysis for the plaque index (PI).

Five clinical trials evaluating the influence of PT additionally to antioxidant therapy in the mean of PI, by analyzed sites, were included in this analysis. Including all studies, the heterogeneity was moderate (*I*^2^ = 47%). Individuals treated with PT plus antioxidants presented a similar mean of PI, per sites, compared to individuals treated with PT only up to one month of follow-up (SMD 0.46 (-0.02, 0.94), *p* = 0.06, *I*^2^ = 74%, *I*^2^*p* = 0.004). However, at three months of follow-up (SMD 0.46 (0.24, 0.68), *p* < 0.0001, *I*^2^ = 0%, *I*^2^*p* = 0.95) and in the pooled results (SMD 0.41 (0.18, 0.64), *p* = 0.0005, *I*^2^ = 47%, *I*^2^*p* = 0.05) individuals treated with PT plus antioxidants presented a lower mean of PI, per sites, compared to individuals treated with PT only (Figure 4).

#### 3.3.3. Meta-Analysis for the Gingival Index (GI)

**Figure 5:**
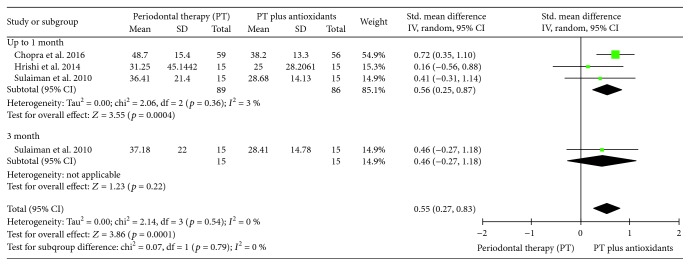
Forest plot of the third meta-analysis for the gingival index (GI).

Six clinical trials evaluating the influence of PT additionally to antioxidant therapy in the mean of GI, per sites, were included in this analysis. Including all studies, the heterogeneity was moderate (*I*^2^ = 43%). At six months or more of follow-up, point individuals treated with PT plus antioxidants presented a similar mean of GI, per sites, compared to individuals treated with PT only (SMD 0.22 (-0.43, 0.88) *p* = 0.50, *I*^2^ = 35%, *I*^2^*p* = 0.22). Up to one month (SMD 0.43 (0.23, 0.63), *p* < 0.0001, *I*^2^ = 0%, *I*^2^*p* = 0.76), up to three months (SMD 0.66 (0.30, 1.02), *p* = 0.0003, *I*^2^ = 56%, *I*^2^*p* = 0.06), and in pooled results (SMD 0.51 (0.31, 0.71) *p* < 0.00001, *I*^2^ = 43%, *I*^2^*p* = 0.05), individuals treated with PT plus antioxidants (*n* = 199) presented a lower mean of GI, per sites, compared to individuals treated with PT only (*n* = 198) (Figure 5).

#### 3.3.4. Meta-Analysis of Bleeding on Probing (BOP)

**Figure 6:**
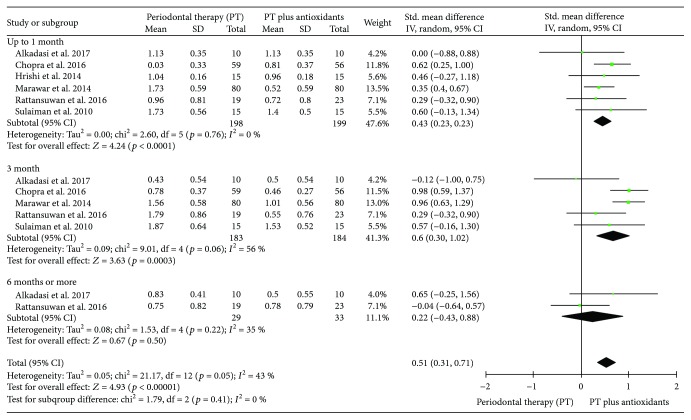
Forest plot of the fourth meta-analysis for bleeding on probing (BOP).

Three clinical trials evaluating the influence of PT plus antioxidants in the mean of BOP per tooth and two per site were included. With the intention of including as many studies as possible, this analysis was conducted with the three studies that evaluate the BOP per tooth and a substantial heterogeneity was observed (*I*^2^ = 64%). During sensitivity analysis, the heterogeneity ranges from 0% to 73%, and to reduce the overall heterogeneity, the three-month follow-up results of Chopra et al. [23] were excluded from the final analysis. Individuals treated with PT plus antioxidants (*n* = 86) presented a lower mean of BOP, per tooth, compared to individuals treated with PT only (*n* = 89) up to one month (SMD 0.56 (0.25, 0.87), *p* = 0.0004, *I*^2^ = 3%, *I*^2^*p* = 0.36) and in pooled results (SMD 0.55 (0.27, 0.83), *p* = 0.0001, *I*^2^ = 0%, *I*^2^*p* = 0.54) (Figure 6).

#### 3.3.5. Meta-Analysis Probing Depth (PD)

**Figure 7:**
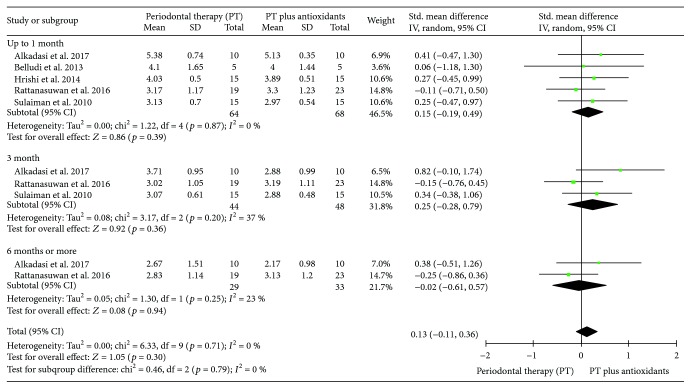
Forest plot of the fifth meta-analysis for probing depth (PD).

Six clinical trials evaluating the influence of PT plus antioxidants in the mean of PD, in millimeters per tooth, were included. The pooled heterogeneity was considerable (*I*^2^ = 70%). During sensitivity analysis, the heterogeneity ranges from 0% to 71%, and in an attempt to reduce the overall and subgrouped heterogeneity, the study of Chopra et al. [23] was excluded from the final analysis. Individuals treated with PT plus antioxidants (*n* = 68) presented a mean of PD similar to individuals treated with PT only (*n* = 64) (SMD 0.13 (-0.11, 0.36), *p* = 0.3, *I*^2^ = 0%, *I*^2^*p* = 0.71), independent of follow-up point evaluated (up to one month (SMD 0.15 (-0.19, 0.49), *p* = 0.39, *I*^2^ = 0%, *I*^2^*p* = 0.87), three months (SMD 0.25 (-0.28, 0.79), *p* = 0.36, *I*^2^ = 37%, *I*^2^*p* = 0.20), or six months or more (SMD -0.02 (-0.61, 0.57), *p* = 0.94, *I*^2^ = 23%, *I*^2^*p* = 0.25)) (Figure 7).

### 3.4. Level of Evidence

To assess the quality of evidence across studies, the GRADE approach was applied. Three different evaluations were performed: overall evaluation of the antioxidant effect regarding periodontal indexes (Table 6), the role of lycopene (Table 7), and the role of green tea (Table 8) in these outcomes. In the overall analysis, a moderate to high quality was observed among the outcomes, in which the flaws presented in the risk of bias were directly associated with the downgrade of the evidence. In lycopene and green tea (GT) evaluation, a moderate certainty and a high certainty were, respectively, detected.

## 4. Discussion

Fifteen clinical trials studies were included in this systematic review, and all of them indicated the beneficial effects of antioxidants during periodontitis treatment. Our meta-analyses showed improvement in the parameters of clinical attachment loss, plaque index, gingival index, and bleeding on probing (except in probing depth); thus, the results may lead to a possible reduction of periodontal inflammation—a pattern among patients. Considering the limitations found in periodontal treatment for complete resolution of the inflammatory process, it is interesting to seek additional adjuvants to the only mechanical treatment.

Furthermore, the complexity of interactions between the microbiota, host, and environment must be considered in periodontal therapy. Therefore, additional strategies on treatment and self-care has to be investigated. Clinically, the reduction of the inflammatory pattern to low levels is important to control the periodontal health state. It is important to mention that the inflammation decrease is essential to establish the health of the periodontal tissues [3]. It is relevant to expose that in patients with severe inflammatory conditions, the reduction of the inflammatory status is essential because it contributes to the planning of periodontal health maintenance or management of reduced periodontium in regenerative therapies [3, 12]. Therefore, because there is an important relationship between the presence of reactive oxygen species and an inflammatory condition, the antioxidant therapy may control the disease.

In this context, etiopathogenic knowledge is fundamental. There are evidences in the literature that point to the role of oxidative stress with a decrease in the antioxidant defenses that stimulate the process of periodontal destruction [34]. The relationship between oxidative stress and periodontal disease is quite strong and can be a two-way path. On one hand, the presence of periodontal inflammation increases the number of oxidative stress markers, and on the other hand, it tends to potentiate aspects of periodontal destruction [35].

The literature supports the hypotheses about the association pathway between antioxidant defense and improved periodontitis. On the other hand, this association needs support and the establishment of a causal relationship by evidence-based clinical decisions regarding the use of antioxidants [5]. In this sense, it is essential to make a broad review of the existing literature for a more accurate picture of this relationship. According to the author, there is not any published article about this systematic review and meta-analysis. It was carried out with the contemporary methodological principles trying to reflect the highest degree of available evidence in this approach.

A systematic review involves the application of methodological strategies that limit bias and evaluate and summarize crucial scientific evidences. These systematic analyzes can help practitioners be aware of the scientific literature [36]. The instruments for quality evaluation and biases of these reviews vary according to the type of study. Besides, systematic reviews may include meta-analysis, in which statistical techniques are used to assess the size of the effect of outcomes. In addition, the level of evidence performed using the GRADE tool enables the elaboration of recommendations for clinical practice [18].

The search strategy used in this study included the most important databases to health science in addition to the PICO's strategy, which allows the comparison of the clinical trial results, verifying if there is an additional effect in the use of antioxidants as adjuvants in conventional PT. The search results show that this approach has been slightly studied, especially in considering the antioxidant diversity, as well as the evaluated parameters. Nevertheless, knowing that the antioxidants used have common objectives, the results can be interpreted as a role of antioxidants in general as adjuvants to conventional PT.

A critical point in the context of this review is the type of outcome evaluated, i.e., in which the studies considered periodontal clinical parameters. It can be observed that the PD was the most used parameter. The PD, in the context of the result of periodontal treatment, is one of the most interesting parameters since it is related to the inflammatory status [3]. Changes in PD are related to changes in clinically detectable inflammation [37]. Thus, taking into account that the analysis of the present study is related to the posttreatment outcome, the outcome seems adequate. Obviously, for the longitudinal evaluation of the outcome of periodontal treatment, the measurement of clinical levels of insertion is fundamental.

However, the literature has pointed out that the absence of clinically detectable inflammation is associated with stability of periodontal destruction [38, 39]. In this sense, the best predictor of periodontal stability is the absence of bleeding on probing [3, 40]. The presence of inflammation is not a good indicator of destruction, but its absence is related to the stoppage of destruction. Therefore, results related to the PD and different forms of evaluation of inflammation, especially with bleeding on probing, are essential. Analysis of marginal inflammation through the GI or its derivatives does not appear to be a suitable parameter. In this review, the importance given by the authors to the markers of inflammation is clear. The plaque evaluation performed in the studies only seems to have the value to verify the effectiveness of self-care because there is no support to affirm the effect of antioxidants on microorganisms of the oral microbiome. Moreover, the patient follow-up through these parameters is important to evaluate the disease course and regression.

Regarding the risk of bias evaluation of the included studies, four studies demonstrated a high risk of bias [24, 26, 27, 33]. Khareava et al. (2016) and Ferial et al. (2018) presented a lack of blinding of both the research participants and the evaluator of the numerical data tabulated between the groups. The blinded experiment in a clinical study helps the reduction of differential assessment of data, changes in interventions applied by the workforce and patients, and the improvement of adherence or retention of study participants, limiting biases during the research [41].

The studies of Elgendy et al. [24] and Manthena et al. [27], besides presenting nonconformities in the blinding, presented a high risk of bias due to the absence of allocation. A detailed description of the method used to generate the random sequence allows to evaluate the possibility of producing comparable groups and reproducing the method in future studies [42].

It is important to emphasize that the quality of the studies presented in this topic is quite impressive, with most studies included presenting a low risk of bias. One of the concerns is the lack of uniformity of the antioxidants used. The number of patients included in the individual studies is quite variable. The use of meta-analyses is aimed at emerging results from different studies, increasing the number of people analyzed, which generates an increase in statistical power.

Considering the problems regarding methodological quality, four studies were excluded from the meta-analysis [24, 26, 27, 33]. Our quantitative synthesis grouped the studies according to the following periodontal clinical outcomes: CAL, BOP, PI, GI, and PD. In addition, we subgrouped in each periodontal analysis the follow-up until 1 month, three months, and six months in order to compare the periodontal condition before and after the therapies studied.

The results of the meta-analyses revealed that using antioxidants as adjuvants to PT improved the following clinical parameters: CAL, BOP, and GI, demonstrating *p* values for the test of significance of the total overall estimate of 0.03, 0.001, and <0.0001, respectively. These data confirm that this therapy has been able to modulate the periodontal tissues to the point of making them clinically healthy due to the absence or very low levels of inflammation. As for PI, the meta-analysis showed a difference with a *p* value for the test of significance of the total overall estimate of 0.0005 and especially 3 months after the therapy (*p* < 0.0001). Of the studies included in this meta-analysis, three presented an antioxidant contact with the dental surface. Thus, this contact may stimulate an astringent effect on plaque [23, 25, 32]. Despite the improvement in the inflammation of patients with periodontitis, the meta-analysis for probing depth obtained a value of 0.30 and showed no statistical difference at any follow-up period.

Generally, the results presented point to more significant reductions in inflammatory parameters when antioxidants are used in comparison to placebo. The GRADE tool showed a moderate to high certainty in the quality of evidence depending not only to the clinical parameter but also to the type of antioxidants used. The clinical significance of the difference between these two therapies should be analyzed warily. However, it is important to note that, regardless of quantity, the reduction of inflammation is significant and deserves attention. Apparently, due to the diversity of the agents used, it is essential that a separate analysis be performed for each of the antioxidants used. The most commonly used antioxidants were lycopene and green tea.

About lycopene, the data showed greater efficacy of this carotenoid in relation to the other antioxidants. The possible explanation for such finding is that this compound has high radical scavenging ability and interferes with other nonoxidizing mechanisms, including anti-inflammatory agents, as reported below [43].

Lycopene belongs to the family of carotenoids, it is an open-chain isomer of *β*-carotene, one of the primary antioxidants in the diet. A natural pigment synthesized by lycopene is responsible for the red color of many fruits and vegetables, such as ripe tomatoes, watermelons, and papayas [44]. Lycopene may accumulate in the lipophilic compartments of the membrane or lipoprotein, thereby being transported by plasma lipoproteins, and the distribution depends on their chemical structure. As a lipophilic compound, lycopene is mainly transported by low-density lipoproteins and can be found in the adrenal gland, liver, prostate, and reproductive tissues (ten times higher than other tissues) [45, 46]. However, the bioavailability of lycopene was higher in the large intestine (57%) than in the small intestine (40%) but its absorption potential was insignificant in the large intestine [47]. The biological effects of lycopene are associated with its antioxidant and nonoxidizing actions, such as anti-inflammatory and cell signalling activities, which are well established. Its antioxidant activity is related to binding to reactive oxygen species (ROS) by different mechanisms: (i) by electron transfer, (ii) by hydrogen atom transfer, or (iii) by adduct formation [48]. Di Mascio et al. reported that lycopene has singlet oxygen quenching ability, showing that it is two times more effective than *β*-carotene, 100 times more potent than *α*-tocopherol, and at least 47 times stronger than vitamin E [49]. In addition, its antioxidant mechanisms are also associated with the elimination of other free radicals, leading to the reduction of intracellular and extracellular ROS levels, and decrease the formation of MDA in plasma and tissue and increase GSH levels and hepatic GSH-Px, SOD, and CAT activities [50]. Also, lycopene also inhibits NF-*κ*B activation, DNA fragmentation, caspase-3 activation, and cytochrome c release [51].

In other ways, it activates the NF-E2 p45-related factor 2 (Nrf2)/HO-1 pathway, by mechanisms of direct interaction of lycopene or its metabolites with the protein cysteine residue Keap1 that induces the expression and translocation of Nrf2, a regulator of expression of antioxidant genes under both physiological and oxidative stress conditions. In addition, it activates kinases that release and translocate Nrf2 to the nucleus [50, 52]. The anti-inflammatory action of lycopene can be attributed mainly to the induction of inflammatory mediators, such as interleukin-1*β* (IL-1*β*), IL-6, IL-8, and tumor necrosis factor-*α* (TNF-*α*) [53].

In the case of GT, its pharmacological effect is mainly due to polyphenols and flavonoids, notably (-) epicatechin (EC), (-) epigallocatechin (EGC), and the derivatives of gallate, such as (−)-epigallocatechin-3-gallate (EGCG), and (−)-epicatechin-3-gallate (ECG), but it has a low concentration of phenolic acids [54]. Several studies have shown that GT has a dubious effect, both antioxidant and prooxidant activities. Its oxidant action can lead to ROS generation, caspase-3 and caspase-9 activation, apoptosis induction, and inhibition of the growth of cancer cells, which is essential in cancer prevention and the oncogenic process. The GT can also inhibit the transcription factor activation, such as NF-*κ*B, activator protein-1 (AP-1), and receptor tyrosine kinase pathways (RTKs). The direct antioxidant activity of GT is related mainly to hydrogen atom transfer, single-electron transfer reactions, and both involving hydroxyl groups, as well as increase in the levels of SOD and catalase that can attenuate lipid peroxidation and protein carbonylation under conditions of oxidative stress [55, 56]. Besides, GT can improve humoral and cellular immunity and has a potent anti-inflammatory effect, inhibiting the level of TNF-*α* [57].

Despite the evidence of the benefits of antioxidants as adjuvants in periodontal therapy, the substantial limitation was the inclusion of a small number of studies in our quantitative synthesis, suggesting more randomized clinical trials which measure periodontal clinical parameters. Another limitation was the use of not only different types of antioxidants between the 15 included studies but also different administrations (capsules, extracts, sachets, dentifrices, gel formulation, etc.), because our review cannot point a specific adjuvant to the clinical treatment.

Moreover, our results corroborate the hypothesis suggested in the scientific literature that oxidative stress is involved in the pathogenesis of periodontal disease. In general, this systematic review indicates that antioxidants are proper adjuvants to periodontal treatment and can improve the oxidative damage promoted to the periodontal tissue during periodontitis.

## 5. Conclusions

Based on clinical trials, this systematic review suggested the use of antioxidants, especially lycopene and GT, as good adjuvants in periodontal therapy, modulating oxidative stress on the periodontium during periodontitis. Therefore, antioxidant therapy may lead to the maintenance of periodontal heath and decrease of inflammatory levels, such as improvement of PI, GI, BOP, and CAL. Further longitudinal studies to better understand the mechanisms of inflammation decrease are highly recommended.

## Figures and Tables

**Table 1:** Search strategy on each electronic database.

Database	Search format
PubMed	(((((((((((((((((((((((((((Chronic Periodontitis[MeSH Terms]) OR Chronic Periodontitis[Title/Abstract]) OR Chronic Periodontitides[Title/Abstract]) OR Periodontitides, Chronic[Title/Abstract]) OR Periodontitis, Chronic[Title/Abstract]) OR Adult Periodontitis[Title/Abstract]) OR Adult Periodontitides[Title/Abstract]) OR Periodontitides, Adult[Title/Abstract]) OR Periodontitis, Adult[Title/Abstract]) OR Periodontal treatment[Title/Abstract]) OR Periodontal therapy[Title/Abstract]) OR nonsurgical periodontal therapy[Title/Abstract]) OR non-surgical periodontal therapy[Title/Abstract]) OR periodontitis[MeSH Terms]) OR periodontitis[Title/Abstract]) OR Periodontitides[Title/Abstract]) OR Pericementitis[Title/Abstract]) OR Pericementitides[Title/Abstract]) OR Periodontal Diseases[MeSH Terms]) OR Periodontal Diseases[Title/Abstract]) OR Disease, Periodontal[Title/Abstract]) OR Diseases, Periodontal[Title/Abstract]) OR Periodontal Disease[Title/Abstract]) OR Parodontosis[Title/Abstract]) OR Parodontoses[Title/Abstract]) OR Pyorrhea Alveolaris[Title/Abstract])) AND ((((((((((((((((((((((((((((((((((((((((((((((Antioxidants[MeSH Terms]) OR Antioxidants[Title/Abstract]) OR Antioxidant Effect[Title/Abstract]) OR Effect, Antioxidant[Title/Abstract]) OR Anti-Oxidant Effect[Title/Abstract]) OR Anti Oxidant Effect[Title/Abstract]) OR Effect, Anti-Oxidant[Title/Abstract]) OR Anti-Oxidant Effects[Title/Abstract]) OR Anti Oxidant Effects[Title/Abstract]) OR Effects, Anti-Oxidant[Title/Abstract]) OR Antioxidant Effects[Title/Abstract]) OR Effects, Antioxidant[Title/Abstract]) OR Resveratrol[Title/Abstract]) OR Tea[MeSH Terms]) OR Tea[Title/Abstract]) OR Green Tea[Title/Abstract]) OR Green Teas[Title/Abstract]) OR Tea, Green[Title/Abstract]) OR Teas, Green[Title/Abstract]) OR Black Tea[Title/Abstract]) OR Black Teas[Title/Abstract]) OR Tea, Black[Title/Abstract]) OR Teas, Black[Title/Abstract]) OR Ascorbic Acid[MeSH Terms]) OR Ascorbic Acid[Title/Abstract]) OR Acid, Ascorbic[Title/Abstract]) OR L-Ascorbic Acid[Title/Abstract]) OR Acid, L-Ascorbic[Title/Abstract]) OR L Ascorbic Acid[Title/Abstract]) OR Vitamin C[Title/Abstract]) OR Hybrin[Title/Abstract]) OR Magnorbin[Title/Abstract]) OR Sodium Ascorbate[Title/Abstract]) OR Ascorbate, Sodium[Title/Abstract]) OR Ascorbic Acid, Monosodium Salt[Title/Abstract]) OR Ferrous Ascorbate[Title/Abstract]) OR Ascorbate, Ferrous[Title/Abstract]) OR Magnesium Ascorbate[Title/Abstract]) OR Ascorbate, Magnesium[Title/Abstract]) OR Magnesium di-L-Ascorbate[Title/Abstract]) OR Magnesium di L Ascorbate[Title/Abstract]) OR di-L-Ascorbate, Magnesium[Title/Abstract]) OR Magnesium Ascorbicum[Title/Abstract]) OR Vitamin E[MeSH Terms]) OR Vitamin E[Title/Abstract])

Scopus	(TITLE-ABS-KEY (“Chronic Periodonti∗”) OR TITLE-ABS-KEY (“Periodontitides, Chronic”) OR TITLE-ABS-KEY (“Periodontitis, Chronic”) OR TITLE-ABS-KEY (“Adult Periodontiti∗”) OR TITLE-ABS-KEY (“Periodontitides, Adult”) OR TITLE-ABS-KEY (“Periodontitis, Adult”) OR TITLE-ABS-KEY (“Periodontal treatment”) OR TITLE-ABS-KEY (“Periodontal therapy”) OR TITLE-ABS-KEY (“nonsurgical periodontal therapy”) OR TITLE-ABS-KEY (“non-surgical periodontal therapy”) OR TITLE-ABS-KEY (Periodontiti∗) OR TITLE-ABS-KEY (Pericementiti∗) OR TITLE-ABS-KEY (“Periodontal Disease∗”) OR TITLE-ABS-KEY (“Disease, Periodontal”) OR TITLE-ABS-KEY (“Diseases, Periodontal”) OR TITLE-ABS-KEY (“Parodontos∗”) OR TITLE-ABS-KEY (“Pyorrhea Alveolaris”)) AND (TITLE-ABS-KEY (Antioxidants) OR TITLE-ABS-KEY (“Antioxidant Effect∗”) OR TITLE-ABS-KEY (“Effect, Antioxidant”) OR TITLE-ABS-KEY (“Anti-Oxidant Effect∗”) OR TITLE-ABS-KEY (“Anti Oxidant Effect∗”) OR TITLE-ABS-KEY (“Effect, Anti-Oxidant”) OR TITLE-ABS-KEY (“Effects, Anti-Oxidant”) OR TITLE-ABS-KEY (“Effects, Antioxidant”) OR TITLE-ABS-KEY (Resveratrol) OR TITLE-ABS-KEY (Tea) OR TITLE-ABS-KEY ("Green Tea∗") OR TITLE-ABS-KEY (“Tea, Green”) OR TITLE-ABS-KEY (“Teas, Green”) OR TITLE-ABS-KEY (“Black Tea∗”) OR TITLE-ABS-KEY (“Tea, Black”) OR TITLE-ABS-KEY (“Teas, Black”) OR TITLE-ABS-KEY (“Ascorbic Acid”) OR TITLE-ABS-KEY (“Acid, Ascorbic”) OR TITLE-ABS-KEY (“L-Ascorbic Acid”) OR TITLE-ABS-KEY (“Acid, L-Ascorbic”) OR TITLE-ABS-KEY (“L Ascorbic Acid”) OR TITLE-ABS-KEY (“Vitamin C”) OR TITLE-ABS-KEY (Hybrin) OR TITLE-ABS-KEY (Magnorbin) OR TITLE-ABS-KEY (“Sodium Ascorbate”) OR TITLE-ABS-KEY (“Ascorbate, Sodium”) OR TITLE-ABS-KEY (“Ascorbic Acid, Monosodium Salt “) OR TITLE-ABS-KEY (“Ferrous Ascorbate”) OR TITLE-ABS-KEY (“Ascorbate, Ferrous”) OR TITLE-ABS-KEY (“Magnesium Ascorbate”) OR TITLE-ABS-KEY (“Ascorbate, Magnesium”) OR TITLE-ABS-KEY (“Magnesium di-L-Ascorbate”) OR TITLE-ABS-KEY (“Magnesium di L Ascorbate”) OR TITLE-ABS-KEY (“di-L-Ascorbate, Magnesium”) OR TITLE-ABS-KEY (“Magnesium Ascorbicum”) OR TITLE-ABS-KEY (“Vitamin E”))

Web of Science	TS=(“Chronic Periodonti∗”) OR TS=(“Periodontitides, Chronic”) OR TS=(“Periodontitis, Chronic”) OR TS=(“Adult Periodontiti∗”) OR TS=(“Periodontitides, Adult”) OR TS=(“Periodontitis, Adult”) OR TS=(“Periodontal treatment”) OR TS=(“Periodontal therapy”) OR TS=(“nonsurgical periodontal therapy”) OR TS=(“non-surgical periodontal therapy”) OR TS=(Periodontiti∗) OR TS=(Pericementiti∗) OR TS=(“Periodontal Disease∗”) OR TS=(“Disease, Periodontal”) OR TS=(“Diseases, Periodontal”) OR TS=(“Parodontos∗”) OR TS=(“Pyorrhea Alveolaris”) AND TS=(Antioxidants) OR TS=(“Antioxidant Effect∗”) OR TS=(“Effect, Antioxidant”) OR TS=(“Anti-Oxidant Effect∗”) OR TS=(“Anti Oxidant Effect∗”) OR TS=(“Effect, Anti-Oxidant”) OR TS=(“Effects, Anti-Oxidant”) OR TS=(“Effects, Antioxidant”) OR TS=(Resveratrol) OR TS=(Tea) OR TS=(“Green Tea∗”) OR TS=(“Tea, Green”) OR TS=(“Teas, Green”) OR TS=(“Black Tea∗”) OR TS=(“Tea, Black”) OR TS=(“Teas, Black”) OR TS=(“Ascorbic Acid”) OR TS=(“Acid, Ascorbic”) OR TS=(“L-Ascorbic Acid”) OR TS=(“Acid, L-Ascorbic”) OR TS=(“L Ascorbic Acid”) OR TS=(“Vitamin C”) OR TS=(Hybrin) OR TS=(Magnorbin) OR TS=(“Sodium Ascorbate”) OR TS=(“Ascorbate, Sodium”) OR TS=(“Ascorbic Acid, Monosodium Salt”) OR TS=(“Ferrous Ascorbate”) OR TS=(“Ascorbate, Ferrous”) OR TS=(“Magnesium Ascorbate”) OR TS=(“Ascorbate, Magnesium”) OR TS=(“Magnesium di-L-Ascorbate”) OR TS=(“Magnesium di L Ascorbate”) OR TS=(“di-L-Ascorbate, Magnesium”) OR TS=(“Magnesium Ascorbicum”) OR TS=(“Vitamin E”)

Cochrane	“Chronic Periodontitis” OR “Chronic Periodontitides” OR “Periodontitides, Chronic” OR “Periodontitis, Chronic” OR “Adult Periodontitis” OR “Adult Periodontitides” OR “Periodontitides, Adult” OR “Periodontitis, Adult” OR “Periodontal treatment” OR “Periodontal therapy” OR “nonsurgical periodontal therapy” OR “non-surgical periodontal therapy” OR “periodontitis” OR “Periodontitides” OR “Pericementitis” OR “Pericementitides” OR “Periodontal Diseases” OR “Disease, Periodontal” OR “Diseases, Periodontal” OR “Periodontal Disease” OR “Parodontosis” OR “Parodontoses” OR “Pyorrhea Alveolaris” AND Antioxidants OR “Antioxidant Effect” OR “Effect, Antioxidant” OR “Anti-Oxidant Effect” OR “Anti Oxidant Effect” OR “Effect, Anti-Oxidant” OR “Anti-Oxidant Effects” OR “Anti Oxidant Effects” OR “Effects, Anti-Oxidant” OR “Antioxidant Effects” OR “Effects, Antioxidant” OR Resveratrol OR TeaOR “Green Tea” OR “Green Teas” OR “Tea, Green” OR “Teas, Green” OR “Black Tea” OR “Black Teas” OR “Tea, Black” OR “Teas, Black” OR “Ascorbic Acid” OR “Acid, Ascorbic” OR “L-Ascorbic Acid” OR “Acid, L-Ascorbic” OR “L Ascorbic Acid” OR “Vitamin C” OR HybrinOR MagnorbinOR “Sodium Ascorbate” OR “Ascorbate, Sodium” OR “Ascorbic Acid, Monosodium Salt” OR “Ferrous Ascorbate” OR “Ascorbate, Ferrous” OR “Magnesium Ascorbate” OR “Ascorbate, Magnesium” OR “Magnesium di-L-Ascorbate” OR “Magnesium di L Ascorbate” OR “di-L-Ascorbate, Magnesium” OR “Magnesium Ascorbicum” OR “Vitamin E”

Lilacs	(tw:((Chronic Periodontitis) OR (Chronic Periodontitides) OR (Periodontitides, Chronic) OR (Periodontitis, Chronic) OR (Adult Periodontitis) OR (Adult Periodontitides) OR (Periodontitides, Adult) OR (Periodontitis, Adult) OR (Periodontal treatment) OR (Periodontal therapy) OR (nonsurgical periodontal therapy) OR (non-surgical periodontal therapy) OR (periodontitis) OR (Periodontitides) OR (Pericementitis) OR (Pericementitides) OR (Periodontal Disease$) OR (Disease, Periodontal) OR (Diseases, Periodontal) OR (Parodontosis) OR (Parodontoses) OR (Pyorrhea Alveolaris))) AND (tw:((Antioxidant$) OR (Antioxidant Effect) OR (Effect, Antioxidant) OR (Anti-Oxidant Effect) OR (Anti Oxidant Effect) OR (Effect, Anti-Oxidant) OR (Effects, Anti-Oxidant) OR (Effects, Antioxidant) OR (Resveratrol) OR (Tea$) OR (Green Tea$) OR (Tea, Green) OR (Teas, Green) OR (Black Tea$) OR (Tea, Black) OR (Teas, Black) OR (Ascorbic Acid) OR (Acid, Ascorbic) OR (L-Ascorbic Acid) OR (Acid, L-Ascorbic) OR (L Ascorbic Acid) OR (Vitamin C) OR (Hybrin) OR (Magnorbin) OR (Sodium Ascorbate) OR (Ascorbate, Sodium) OR (Ascorbic Acid, Monosodium Salt) OR (Ferrous Ascorbate) OR (Ascorbate, Ferrous) OR (Magnesium Ascorbate) OR (Ascorbate, Magnesium) OR (Magnesium di-L-Ascorbate) OR (Magnesium di L Ascorbate) OR (di-L-Ascorbate, Magnesium) OR (Magnesium Ascorbicum) OR (Vitamin E)))

Google Scholar	Periodontitis+ Antioxidants -review

OpenGrey	Periodontitis AND Antioxidants

**Table 2:** Criteria for risk assessment of bias according to “the Cochrane Collaboration's tool for assessing risk of bias (Higgins et al., 2011).

Random sequence generation	
Criteria for judgment of “low risk” of bias	The articles that appropriately described the method of randomization
Criteria for judgment of “high risk” of bias	Articles that presented a methodological failure in the randomization criterion or the difficult reproducibility method
Criteria for judgment of “unclear risk” of bias	When the articles did not describe the method of randomization

Allocation concealment	
Criteria for judgment of “low risk” of bias	When the allocation sequences of samples were concealed in the randomization
Criteria for judgment of “high risk” of bias	When the sequences of allocation of samples were not concealed at randomization
Criteria for judgment of “unclear risk” of bias	When the allocation sequences were unreported

Blinding of participants and researchers	
Criteria for judgment of “low risk” of bias	When the sample was blind
Criteria for judgment of “high risk” of bias	If the methodology could not be blinded for whatever reason (sample/appraiser)
Criteria for judgment of “unclear risk” of bias	When the sample was not reported either way

Blinding of outcome assessment	
Criteria for judgment of “low risk” of bias	When the evaluators reported that the blinding in the evaluation was effective
Criteria for judgment of “high risk” of bias	If the study informed the evaluators how the blinding was done
Criteria for judgment of “unclear risk” of bias	When the blinding was not reported

Incomplete outcome data	
Criteria for judgment of “low risk” of bias	When there was an exhaustive description of the main data
Criteria for judgment of “high risk” of bias	If there was a loss due to an incomplete description of the main results regardless of quantity, nature, and manipulation
Criteria for judgment of “unclear risk” of bias	When the results were not reported

Selective reporting	
Criteria for judgment of “low risk” of bias	When the discussion excluded some of the results
Criteria for judgment of “high risk” of bias	When the article discussed the data completely
Criteria for judgment of “unclear risk” of bias	When the organization of the results in the discussion was unclear

**Table 3:** Risk of bias evaluation of nonrandomized clinical trials according to the ROBINS-I tool [15].

Domain of bias	Description
Preintervention	
Bias due to confounding	Baseline confounding. When one or more preintervention prognostic factors predict the intervention received at baseline (start of follow-up)
	Time-varying confounding. When the intervention received can change over time and when postintervention prognostic factors affect the intervention received after baseline
Bias in selecting participants for study	When selection of participants is related to both intervention and outcome
	Lead time bias. When some follow-up time is excluded from the analysis
	Immortal time bias. When the interventions are defined in such a way that there is a period of follow-up during which the outcome cannot occur

At intervention	
Bias in classifying interventions	When intervention status is misclassified
	Nondifferential misclassification. Is unrelated to the outcome
	Differential misclassification. Is related to the outcome or to the risk of the outcome

Postintervention	
Bias due to deviating from intended intervention	When there are systematic differences between intervention and comparator groups in the care provided
Bias due to missing data	When attrition (loss to follow-up), missed appointments, incomplete data collection, and exclusion of participants from analysis by primary investigators occur
Bias in measuring outcomes	When outcomes are misclassified or measured with error
	Nondifferential measurement error. Is unrelated to the intervention received; it can be systematic or random
Bias in selecting reported result	Selective reporting of results that should be sufficiently reported to allow the estimate to be included in a meta-analysis (or other synthesis) is considered. When selective reporting is based on the direction, magnitude, or statistical significance of intervention effect estimates. Selective outcome reporting. When the effect estimate for an outcome measurement was selected from among analyses of multiple outcome measurements for the outcome domain. Selective analysis reporting. When results are selected from intervention effects estimated in multiple ways

Judgment for each domain	
Low RoB	Study is comparable to a well-performed, randomized trial with regard to this domain
Moderate RoB	Study is sound for a nonrandomized study with regard to this domain but cannot be considered comparable to a well-performed, randomized trial
Serious RoB	Study has some important problems in this domain
Critical RoB	Study is too problematic in this domain to provide any useful evidence on the effects of intervention
No information	No information on which to base a judgment about risk of bias for this domain

Overall judgment	
Low RoB	Study is judged to be at low risk of bias for all domains
Moderate RoB	Study is judged to be at low or moderate risk of bias for all domains
Serious RoB	Study is judged to be at serious risk of bias in at least one domain, but not at critical risk of bias in any domain
Critical RoB	Study is judged to be at critical risk of bias in at least one domain
No information	No clear indication that the study is at serious or critical risk of bias, and there is a lack of information in one or more key domains of bias (a judgment is required for this)

**Table 4:** Summary of the included studies.

Author (year)	Study design	Sample description	Periodontitis diagnostic method		Periodontal treatment	Treatment antioxidant	Statistical analysis	Outcome
		Sample size and source, age (years), gender, and groups	Clinical	Laboratory				
Alkadasi et al. (2017)	RCT	*n* = 20, Cairo, Egypt Age: 35-58 Males: 14 Females:16 Control: 10 Intervention: 10	PI GI PPD CAL	sRANKL levels in GCF	Scaling and root planning and modified Widman flap procedure	*N*-Acetylcysteine (NAC) capsules (600 mg; Swanson Health Products Co., Fargo, ND, USA)	One-way ANOVA with post Dunnett tests	The use of adjunctive NAC resulted in a significant reduction in probing depths in the S-NAC group when compared to the S-nonNAC group at 3 months, but no statistically significant differences in GCF sRANKL levels were observed in the sites that underwent surgical treatment with or without NAC at different time intervals.

Alkadasi et al. (2017)	RCT	*n* = 42, Andhra Pradesh, India Age: 25-52 Males: 21 Females:21 Control: 21 Intervention: 21	PI MGI PPD CAL BOP	Salivary interleukin 1 beta (IL-1*β*) and uric acid; serum tumor necrosis factor alpha (TNF-*α*)	Scaling and root planning	Lycopene (8 mg/day, LycoRed, JAGSONPAL Pharmaceuticals)	Paired *t*-test for intragroup and Student's independent *t*-test for intergroup; ANCOVA	There was a significant improvement in the parameters of MGI, PI, BOP, IL-1*β*, and salivary uric acid, but the improvement in PPD and CAL parameters was not statistically significant.

Arora et al. (2013)	RCT	*n* = 40, Ahvaz, Iran Males: 21 Females: 19 Age: 25-57 Control: 20 Intervention: 20	PPD	Antioxidant capacity (TAC), malondialdehyde (MDA), total cholesterol (TC), triglycerides (TG), and high-density lipoprotein cholesterol (HDL-C)	Scaling and root planning	Chicory leaf extract (2 g/day)	*t*-test independent	Chicory leaf extract with nonsurgical periodontal therapy may be helpful in controlling the periodontal status.

Babaei et al. (2018)	RCT	*n* = 10, source: UI Age: >10 Gender: UI Control: 5 Intervention: 5	PPD CAL BOP	—	Scaling and root planning	Lycopene (4 mg/day; Lycotas Pharma. Co.)	Paired *t*-test and independent Student's *t*-test	Results show that lycopene is a promising treatment modality as an adjunct to full-mouth SRP of the oral cavity in patients with moderate periodontal disease.

Belludi et al. (2013)	RCT	*n* = 120, Manipal, Karnataka, India Age: 20-50 Males: 61 Females: 54 Control: 59 Intervention: 56	GI PI PPD CAL BI	Total antioxidant capacity in GCF and plasma	Scaling and root planning	Sachets containing green tea (240 ml of water)	Repeated measures ANOVA with post hoc Bonferroni test; independent sample *t*-test	Green tea intake as a component of nonsurgical periodontal therapy is promising for superior and rapid resolution of the disease process. Green tea increases the total antioxidant capacity of GCF and plasma along with potent anti-inflammatory, astringent, and antiplaque effects.

Chopra et al. (2016)	RCT	*n* = 40, Tanta, Egypt Age: 30-60 Males: 21 Females:19 Control: 20 Intervention: 20	PI GI PPD CAL	Pentraxin-3 (PXT3) levels in GCF	Scaling and root planning	Gel formulation was prepared from tea tree oil (TTO) (5%, Melaleuca alternifolia, Sigma®, Steinheim, Germany)	Paired *t*-test and independent Student's *t*-test	The local delivery of TTO gel in case of chronic periodontitis may have some beneficial effects to augment the results of the conventional periodontal therapy.

Elgendy et al. (2013)	RCT	*n* = 30, Tehran, Iran Males:10 Females: 20 Control: 15 Intervention: 15	PPD BI PI	—	Scaling and root planning (SRP)	Green tea (Lahijan green tea)	Wilcoxon test; Mann–Whitney *U* test	The results show that PD and BI reduced significantly in both groups before and after SRP; this reduction in the intervention group was higher than the control group.

Ferial et al. (2018)	RCT pilot	*n* = 30, Manipal, India Age: 18-60 Males: 13 Females: 17 Control: 15 Intervention: 15	GI PI BOP PPD CAL	Parameters of total antioxidant capacity (TOAC) and glutathione-S-transferase (GST) in GCF	Scaling and root planning	Green tea dentifrice (1,4%)	Paired *t*-test and independent Student's *t*-test, Wilcoxon test, and Mann–Whitney *U* test	Green tea dentifrice use showed statistically significant improvements in GI, BOP, CAL, TAOC, and GST levels on intra- and intergroup comparisons at 4 weeks. The results of the present study assert the use of green tea dentifrice as an adjunct to SRP during the active and healing phases following periodontal therapy, thereby enhancing the clinical outcomes.

Hrishi et al. (2016)	Open RCT	*n* = 84, Nalchik, Russia Age: 38-62 Males: 39 Females: 45 Control: 45 Intervention: 39	GI PI BOP PMA	Nitrite/nitrate levels, interleukin-1*β*; (IL-1*β*), interleukin-6 (IL-6), interleukin-10 (IL-10) in GCF	Scaling and root planning. In addition, application of chlorhexidine 0.06%	Standardized fermented papaya gel (SFPG, 7 g)	Mann–Whitney *U* test	All parameters showed a significant improvement in the comparison of the test group with the control group.

Kharaeva et al. (2016)	RCT	*n* = 30, Chinoutpalli, India Age: 18-35 Male: 16 Female: 14 Control: 15 Intervention: 15	PI GI PPD	—	Scaling and root planning	CoQ10 (Qute 120 mg by Yash Pharma International)	Paired *t*-test and independent Student's *t*-test	The use of coenzyme Q10 oral supplements as an adjunct to scaling and root planning showed significant reduction in gingival inflammation when compared to scaling and root planning alone.

Manthena et al. (2015)	RCT	*n* = 160, Loni, Ahmednagar Age: 35-58 Gender: UI Control: UI Intervention: UI	GI PDI CPI	—	Scaling and root planning	Tablet melatonin 3 mg daily at night	*Z*-test	There was significant improvement in all the indices in the group test as compared to the control group. Melatonin is a potential antioxidant, and the clinical improvement it showed was significantly superior to that of the standard control group.

Marawar et al. (2014)	Clinical trial	*n* = 20, Udaipur, Rajasthan Age: 30-60 Gender: UI Control: 10 Intervention: 10	Community periodontal index of treatment needs	Uric acid determination in saliva sample	Scaling and root planning	Lycopene softgel (6 mg/dose)	One-way ANOVA	There was a reduction in periodontal inflammation with an increase in the salivary uric acid levels seen in subgroups treated by antioxidants in both gingivitis and periodontitis groups.

Mathur et al. (2013)	RCT	*n* = 48, Bangkok, Thailand Age: 37-74 Males: 22 Females:20 Control: 23 Intervention: 19	PPD CAL GI BOP	—	Scaling and root planning	Green tea gel (12% *w*/*w* of green tea—*C. sinensis* extract)	Regression test; Mann–Whitney *U* test; Friedman's test	Green tea gel could provide a superior benefit in reducing BOP and gingival inflammation when used as an adjunct to nonsurgical periodontal treatment.

Rattanasu et al. (2016)	RCT	*n* = 60, Haryana, India Age: >18 Control: 22 (male : female: 3 : 8) Intervention: 38 (male : female: 4 : 1)	PI GI BOP PPD CAL	Levels of superoxide dismutase (SOD) activity (%)	Scaling and root planning	Vitamin E softgel (200 mg/day)	Mann–Whitney *U* test	Systemic and local SOD levels are lowered in periodontitis. Adjunctive vitamin E supplementation improves periodontal healing as well as antioxidant defense.

Singh et al. (2014)	RCT	*n* = 30, Damascus, Syria Age: 23-65 Male: 18 Female: 42 Control:15 Intervention: 15	PPD CAL BOP PI GI	Parameters of/total antioxidant capacity (TOAC) in plasma	Scaling and root planning	Vitamin C	Paired *t*-test and independent Student's *t*-test; Mann–Whitney *U* test	The nonsurgical periodontal therapy seems to reduce the oxidative stress during the periodontal inflammation.

RCT: randomized controlled trial; PI: plaque index; GI: gingival index; PD: probing depth; CAL: clinical attachment loss; GCF: gingival crevicular fluid; MGI: modified gingival index; BOP: bleeding on probing; UI: unreported information; BI: percentage of sites with bleeding on probing; PMA: Parma's papillae-gum margin-alveolar; CPI: community periodontal index.

**Table 5:** Risk of bias evaluation in nonrandomized trials (ROBINS-I tool).

Domains								
Author	Preintervention		At intervention	Postintervention				Overall risk of bias judgment
	Bias due to confounding	Bias in selecting participants for the study	Bias in classifying interventions	Bias due to deviations from intended intervention	Bias due to missing data	Bias in measuring outcomes	Bias in selecting reported result	
Mathur et al. (2013)	Low	Low	Low	Low	NI	Low	Low	Low

The categories for risk of bias judgements are “low risk,” “moderate risk,” “serious risk,” and “critical risk” of bias. “Low risk” corresponds to the risk of bias in a high-quality randomized trial; NI: no information on which to base a judgement about risk of bias for this domain.

**Table 6:** Antioxidant effect regarding periodontal indexes.

Antioxidants compared to placebo for periodontal therapy					
Patient or population: periodontal therapy Setting: Intervention: antioxidants Comparison: placebo					

Outcomes	Anticipated absolute effects^∗^ (95% CI)		Relative effect (95% CI)	No. of participants (studies)	Certainty of the evidence (grade)
	Risk with placebo	Risk with antioxidants			

Reduction of bleeding on probing assessed with the bleeding on probing index Follow-up: 3 months	940 per 1.000	0 per 1.000 (0 to 0)	Cannot be estimated	163 (3 RCTs)	⨁⨁⨁◯ Moderate^a^
Reduction of the plaque index assessed with the Silness and Loe plaque index Follow-up: 3 months	756 per 1.000	0 per 1.000 (0 to 0)	Cannot be estimated	243 (5 RCTs)	⨁⨁⨁⨁ High
Improvement of the gingival index assessed with the Loe and Silness gingival index Follow-up: 3 months	1.000 per 1.000	0 per 1.000 (0 to 0)	Cannot be estimated	291 (6 RCTs)	⨁⨁⨁◯ Moderate^b^
Improvement of clinical attachment loss (improvement of CAL) assessed with clinical attachment loss, in mm Follow-up: 3 months	720 per 1.000	0 per 1.000 (0 to 0)	Cannot be estimated	261 (5 RCTs)	⨁⨁⨁⨁ High
Improvement of probing depth assessed with probing depth, in mm. Follow-up: 3 months	695 per 1.000	0 per 1.000 (0 to 0)	Cannot be estimated	253 (6 RCTs)	⨁⨁⨁◯ Moderate^b^

^∗^The risk in the intervention group (and its 95% confidence interval) is based on the assumed risk in the comparison group and the relative effect of the intervention (and its 95% CI). CI: confidence interval. GRADE working group grades of evidence. High certainty: we are very confident that the true effect lies close to that of the estimate of the effect. Moderate certainty: we are moderately confident in the effect estimate: the true effect is likely to be close to the estimate of the effect, but there is a possibility that it is substantially different. Low certainty: our confidence in the effect estimate is limited: the true effect may be substantially different from the estimate of the effect. Very low certainty: we have very little confidence in the effect estimate: the true effect is likely to be substantially different from the estimate of the effect. ^a^The absence of information regarding the randomization process in Belludi et al., 2013. ^b^Half of the studies presented selection bias. Comments.

**Table 7:** The role of lycopene.

Summary of findings					
Lycopene compared to placebo for periodontal therapy					
Patient or population: periodontal therapy Setting: Intervention: lycopene Comparison: placebo					

Outcomes	Anticipated absolute effects^∗^ (95% CI)		Relative effect (95% CI)	No. of participants (studies)	Certainty of the evidence (grade)
	Risk with placebo	Risk with lycopene			

Reduction in bleeding on probing assessed with the bleeding on probing index Follow-up: 1 month	808 per 1.000	0 per 1.000 (0 to 0)	Cannot estimated	52 (2 RCTs)	⨁⨁⨁◯ Moderate^a^
Improvement of clinical attachment loss assessed with clinical attachment loss, in mm. Follow-up: 1 month	192 per 1.000	0 per 1.000 (0 to 0)	Cannot be estimated	52 (2 RCTs)	⨁⨁⨁◯ Moderate^a^
Improvement on probing depth assessed with probing depth Follow-up: 1 month	0 per 1.000	0 per 1.000 (0 to 0)	Cannot be estimated	52 (2 RCTs)	⨁⨁⨁◯ Moderate^a^

GRADE working group grades of evidence. High certainty: we are very confident that the true effect lies close to that of the estimate of the effect. Moderate certainty: we are moderately confident in the effect estimate: the true effect is likely to be close to the estimate of the effect, but there is a possibility that it is substantially different. Low certainty: our confidence in the effect estimate is limited: the real effect may be substantially different from the estimate of the effect. Very low certainty: we have very little confidence in the effect estimate: the true effect is likely to be substantially different from the estimate of the effect. ∗The risk in the intervention group (and its 95% confidence interval) is based on the assumed risk in the comparison group and the relative effect of the intervention (and its 95% CI); CI: confidence interval). Comments.

**Table 8:** The role of green tea.

Summary of findings					
Green tea compared to placebo for periodontal therapy					
Patient or population: periodontal therapy Setting: Intervention: green tea Comparison: placebo					

Outcomes	Anticipated absolute effects^∗^ (95% CI)		Relative effect (95% CI)	No. of participants (studies)	Certainty of the evidence (grade)
	Risk with placebo	Risk with green tea			

Reduction on bleeding on probing assessed with the bleeding on probing index Follow-up: 1 month	203 per 1.000	0 per 1.000 (0 to 0)	Cannot be estimated	145 (2 RCTs)	⨁⨁⨁⨁ High
Reduction on the plaque index assessed with the Silness and Loe plaque index Follow-up: 1 month	0 per 1.000	0 per 1.000 (0 to 0)	Cannot be estimated	145 (2 RCTs)	⨁⨁⨁⨁ High
Reduction in gingival index assessed with the Loe and Silness gingival index Follow-up: 1 month	1.000 per 1.000	0 per 1.000 (0 to 0)	Cannot be estimated	145 (2 RCTs)	⨁⨁⨁⨁ High
Improvement in clinical attachment loss assessed with clinical attachment loss, in mm. Follow-up: 1 month	1.000 per 1.000	0 per 1.000 (0 to 0)	Cannot be estimated	145 (2 RCTs)	⨁⨁⨁⨁ High
Improvement on probing depth assessed with probing depth, in mm. Follow-up: 1 month	0 per 1.000	0 per 1.000 (0 to 0)	Cannot estimated	145 (2 RCTs)	⨁⨁⨁⨁ High

GRADE working group grades of evidence. High certainty: we are very confident that the true effect lies close to that of the estimate of the effect. Moderate certainty: we are moderately confident in the effect estimate: the true effect is likely to be close to the estimate of the effect, but there is a possibility that it is substantially different. Low certainty: our confidence in the effect estimate is limited: the true effect may be substantially different from the estimate of the effect. Very low certainty: we have very little confidence in the effect estimate: the true effect is likely to be substantially different from the estimate of the effect. ∗The risk in the intervention group (and its 95% confidence interval) is based on the assumed risk in the comparison group and the relative effect of the intervention (and its 95% CI). CI: confidence interval. Comments.
